# Closed-loop insulin delivery in pregnant women with type 1 diabetes (CRISTAL): a multicentre randomized controlled trial – study protocol

**DOI:** 10.1186/s12884-023-05481-0

**Published:** 2023-03-16

**Authors:** Kaat Beunen, Nancy Van Wilder, Dominique Ballaux, Gerd Vanhaverbeke, Youri Taes, Xavier-Philippe Aers, Frank Nobels, Joke Marlier, Dahae Lee, Joke Cuypers, Vanessa Preumont, Sarah E. Siegelaar, Rebecca C. Painter, Annouschka Laenen, Pieter Gillard, Chantal Mathieu, Katrien Benhalima

**Affiliations:** 1 Clinical and Experimental Endocrinology, Department of Chronic Diseases and Metabolism, KU Leuven, Herestraat 49, Leuven, 3000 Belgium; 2 Department of Endocrinology, University Hospital Brussels, Laarbeeklaan 101, Jette, 1090 Belgium; 3 Department of Endocrinology, Vitaz Campus Sint-Niklaas Moerland, Moerlandstraat 1, Sint-Niklaas, 9100 Belgium; 4 Department of Endocrinology, General Hospital Groeninge Kortrijk, President Kennedylaan 4, Kortrijk, 8510 Belgium; 5 Department of Endocrinology, General Hospital Sint-Jan Brugge, Ruddershove 10, Brugge, 8000 Belgium; 6 Department of Endocrinology, General Hospital Delta Campus Rumbeke, Deltalaan 1, Roeselare, 8800 Belgium; 7 Department of Endocrinology, OLV Hospital Aalst, Moorselbaan 164, Aalst, 9300 Belgium; 8 Department of Endocrinology, Ghent University Hospital, Corneel Heymanslaan 10, Gent, 9000 Belgium; 9 Department of Endocrinology, Imelda Hospital Bonheiden, Imeldalaan 9, Bonheiden, 2820 Belgium; 10 Department of Endocrinology, General Hospital Turnhout Campus Sint-Jozef, Steenweg op Merksplas 44, Turnhout, 2300 Belgium; 11 Department of Endocrinology, University Hospital Saint-Luc, Avenue Hippocrate 10, Brussel, 1200 Belgium; 12 Department of Endocrinology and Metabolism, Amsterdam University Medical Centres, Meibergdreef 9, Amsterdam, 1105 the Netherlands; 13 Amsterdam Gastroenterology Endocrinology and Metabolism, Amsterdam University Medical Centres, Amsterdam, the Netherlands; 14 Department of Obstetrics & Gynecology, Amsterdam University Medical Centres, De Boelelaan, Amsterdam, the Netherlands; 15 Amsterdam Reproduction and Development, Amsterdam University Medical Centres, Amsterdam, The Netherlands; 16 Center of Biostatics and Statistical bioinformatics, KU Leuven, Kapucijnenvoer 35 bloc d – box 7001, Leuven, 3000 Belgium; 17 Department of Endocrinology, University Hospitals Gasthuisberg Leuven, Herestraat 49, Leuven, 3000 Belgium

**Keywords:** Type 1 diabetes mellitus, Pregnancy, Closed-loop insulin delivery, Automated insulin delivery, Artificial pancreas

## Abstract

**Background:**

Despite increasing use of continuous glucose monitoring (CGM) and continuous subcutaneous insulin infusion (CSII, insulin pumps) in type 1 diabetes (T1D) in pregnancy, achieving recommended pregnancy glycaemic targets (3.5–7.8 mmol/L or 63–140 mg/dL) remains challenging. Consequently, the risk of adverse pregnancy outcomes remains high. Outside pregnancy, hybrid closed-loop (HCL) insulin delivery systems have led to a paradigm shift in the management of T1D, with 12% higher time in glucose target range (TIR) compared to conventional CSII. However, most commercially available HCL systems are currently not approved for use in pregnancy. This study aims to evaluate the efficacy, safety and cost-effectiveness of the MiniMed™ 780G HCL system (Medtronic) in T1D in pregnancy.

**Methods:**

In this international, open-label, randomized controlled trial (RCT), we will compare the MiniMed™ 780G HCL system to standard of care (SoC) in T1D in pregnancy. Women aged 18–45 years with T1D diagnosis of at least one year, HbA1c ≤ 86 mmol/mol (≤ 10%), and confirmed singleton pregnancy up to 11 weeks 6 days will be eligible. After providing written informed consent, all participants will wear a similar CGM system (Guardian™ 3 or Guardian™ 4 CGM) during a 10-day run-in phase. After the run-in phase, participants will be randomised 1:1 to 780G HCL (intervention) or SoC [control, continuation of current T1D treatment with multiple daily injections (MDI) or CSII and any type of CGM] stratified according to centre, baseline HbA1c (< 53 vs. ≥ 53 mmol/mol or < 7 vs. ≥ 7%), and method of insulin delivery (MDI or CSII). The primary outcome will be the time spent within the pregnancy glucose target range, as measured by the CGM at four time points in pregnancy: 14–17, 20–23, 26–29, and 33–36 weeks. Prespecified secondary outcomes will be overnight TIR, time below range (TBR: <3.5 mmol/L or < 63 mg/dL), and overnight TBR. Other outcomes will be exploratory. The planned sample size is 92 participants. The study will end after postpartum discharge from hospital. Analyses will be performed according to intention-to-treat as well as per protocol.

**Discussion:**

This large RCT will evaluate a widely used commercially available HCL system in T1D in pregnancy. Recruitment began in January 2021 and was completed in October 2022. Study completion is expected in May 2023.

**Trial registration:**

ClinicalTrials.gov: NCT04520971. Registration date: August 20, 2020. https://clinicaltrials.gov/ct2/show/NCT04520971

## Background

Pregnancies complicated by type 1 diabetes (T1D) are associated with an increased risk of adverse pregnancy outcomes such as congenital anomalies, miscarriages, preeclampsia, preterm delivery, large-for-gestational age (LGA) infants, and perinatal mortality [1–4]. To reduce the risk of these complications, women with T1D are advised to achieve near normoglycaemia before and during pregnancy, with a time in range (TIR) of 3.5–7.8 mmol/L (63–140 mg/dL) of > 70% [5]. Unfortunately, given the gestational variability in insulin sensitivity and absorption, achieving and maintaining tight glycaemic control in pregnancy is often difficult to accomplish, even with increasing use of new diabetes technologies including continuous glucose monitoring (CGM) and continuous subcutaneous insulin infusion (CSII, insulin pumps) [6–9].

Studies have shown that both multiple daily injections (MDI) and insulin pumps are effective treatment approaches for T1D in pregnancy [10–14]. However, insulin pumps offer advantages in terms of flexibility in insulin basal rates and bolusing, facilitating adaptation to the high glycaemic variability in early pregnancy and timely adjustment to the increasing insulin need later in pregnancy. Hybrid closed-loop (HCL) insulin delivery systems provide automated glucose-responsive insulin delivery overnight, between meals and additional manually triggered premeal boluses. In children, adolescents as well as adults, HCL systems have led to a paradigm shift in T1D management with better glycaemic control, less hypoglycaemia frequency and duration, and better quality of life [15–17]. HCL systems hold promise in the management of T1D in pregnancy as shown by smaller studies [18–23]. However, most commercially available HCL systems are currently not approved for use in pregnancy, and there could be concerns regarding the HCL algorithms’ ability to adapt to the rapidly changing insulin requirements typical to pregnancy. Larger, longer-duration multicentre studies are therefore needed to assess whether commercially available HCL systems can safely improve glycaemic control in T1D in pregnancy and improve pregnancy outcomes.

This study aims to evaluate the safety, efficacy, feasibility and cost-effectiveness of the MiniMed™ 780G HCL system (Medtronic) compared to standard of care (SoC) in pregnant women with T1D. We hypothesize that the 780G HCL system in T1D in pregnancy can improve glycaemic control with less hypoglycaemia, both at night and during the day. This, in turn, might improve pregnancy outcomes.

## Methods

### Study design and setting

**Fig. 1:**
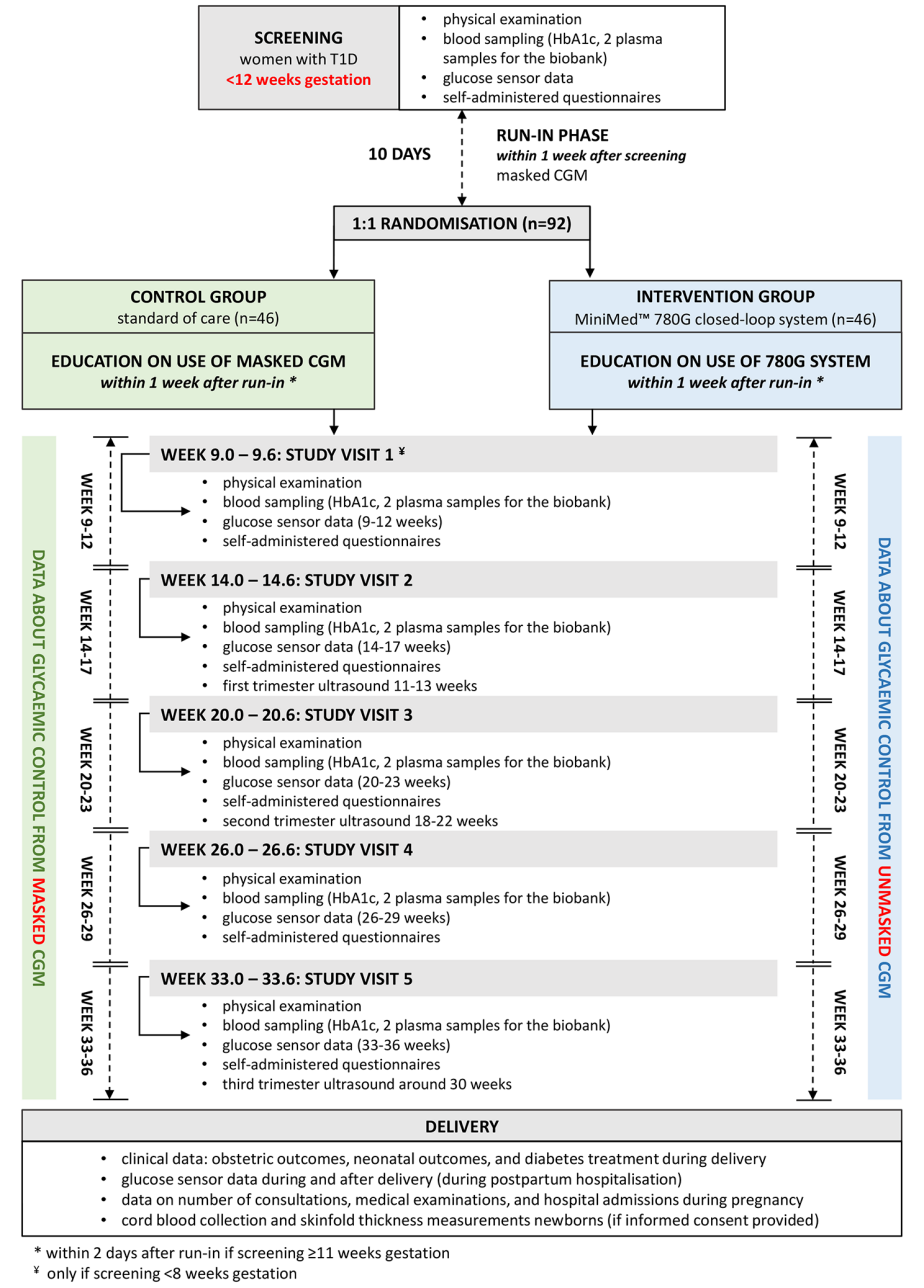
The CRISTAL study flow diagram T1D type 1 diabetes; HbA1c glycated haemoglobin; CGM continuous glucose monitoring

The CRISTAL study is an open-label, multicentre randomized controlled trial (RCT) with the participation of 12 hospitals in Belgium and the Netherlands comparing the 780G HCL system with SoC in T1D in pregnancy. Pregnant women with T1D, diagnosed at least one year before study participation, and a glycated haemoglobin (HbA1c)  ≤ 86 mmol/mol (≤ 10%) will be eligible for study participation, providing their pregnancy has not progressed beyond 11 weeks and 6 days. After providing written informed consent, all participants will be asked to wear a similar (masked) CGM system [Guardian™ Link (3) transmitter with Guardian™ Sensor (3), or the Guardian™ 4 transmitter with Guardian™ 4 sensor] during a 10-day run-in phase. Masking is not required for participants already using the Guardian™ 3 or 4 CGM before the run-in phase. After the run-in phase, participants will be randomised 1:1 to the 780G HCL system (intervention) or to SoC (control, continuation of current T1D treatment approved for use in pregnancy). In both randomisation groups, the glucose target will be set to 3.5–7.8 mmol/L (63–140 mg/dL), with the aim of a TIR > 70%. Differences in glycaemia between both randomisation groups will be evaluated by similar CGM data collected during 21 days at different time points during pregnancy: at 9–12 (if inclusion < 8 weeks), 14–17, 20–23, 26–29, and 33–36 weeks. The Guardian™ 3 CGM will be masked for participants in the control group who routinely use a different glucose monitoring method. Standardised procedures (within the field of endocrinology and obstetrics) will be used for management and follow-up of all participants across all centres, independent of the randomisation group. The study will end after postpartum discharge from hospital. After delivery, the 780G HCL system can be continued in line with normal routine. An overview of the study is shown in Fig. 1. The study has been registered in ClinicalTrials.gov as NCT04520971. The study protocol was approved by the Medical Ethics Review Committees of all participating centres as well as the national competent authorities (Belgian registration number: B3222020000272; Dutch registration number NL78535.000.21).

### Modifications to the study protocol

To facilitate recruitment, three additional centres participated: University Hospital Saint-Luc, General Hospital Turnhout Campus Sint-Jozef, and Amsterdam University Medical Centres location AMC. This has led to an amendment of the original study protocol (last approved version: fourth version, dd 14-10-2021). In addition, the Guardian™ 4 CGM was launched and received CE mark in May 2021. The Guardian™ 4 sensor has a new advanced sensor calibration algorithm but is otherwise similar to the Guardian™ 3 sensor, and is intended to minimize or eliminate required calibrations, thereby reducing the glucose management burden for the user. The Guardian™ 4 sensor is physically identical to the Guardian™ 3 sensor, and the Guardian™ 4 transmitter is equivalent to the Guardian Connect Transmitter with the addition of the G Algorithm, indicating the equivalency between the Guardian™ 3 and Guardian™ 4 CGM (data on files). Therefore, no significant biases are expected when switching from the Guardian™ 3 CGM to the Guardian™ 4 CGM in the intervention group, nor to compare data with the (masked) Guardian™ 3 CGM used during the run-in phase or when used in the control group.

### Objectives

This research answers the need to evaluate whether the 780G HCL system in pregnant women with T1D can improve glycaemic control with less hypoglycaemia compared to SoC. This, in turn, might improve pregnancy outcomes in women with T1D. All outcomes of the core outcome set (COS) for RCTs evaluating the effectiveness of interventions for the treatment of pregnant women with pregestational diabetes mellitus will be reported [24].

#### *Primary outcome*

The primary outcome will be the percentage of time spent within the T1D in pregnancy glycaemic target range (TIR) of 3.5–7.8 mmol/L (63–140 mg/dL) as measured by the (masked) CGM at four specified time points in pregnancy (14–17, 20–23, 26–29, and 33–36 weeks). TIR was chosen as primary outcome since an increase in TIR has been associated with less complications both in pregnant and non-pregnant people with T1D [9, 25].

#### *Secondary outcomes*

Three prespecified secondary outcomes as measured by the (masked) CGM at the four specified time points in pregnancy will be:

1. % overnight (midnight till 6 am) TIR;
2. % time below range (TBR) (< 3.5 mmol/L or < 63 mg/dL);
3. % overnight TBR.

#### Other secondary outcomes will be exploratory.

- Maternal glycaemic outcomes

% TIR during the day (6 am till midnight); % (overnight) TIR 9–12 weeks; mean sensor glucose level; % (overnight) time above range [TAR > 7.8 mmol/L (> 140 mg/dL)]; % time spent > 10 mmol/L (> 180 mg/dL), < 3.9 mmol/L (< 70 mg/dL), < 3.3 mmol/L (< 60 mg/dL), < 3.0 mmol/L (< 54 mg/dL), and < 2.8 mmol/L (< 50 mg/dL); % time in non-pregnant target range 3.9–10 mmol/L (70–180 mg/dL); low blood glucose Index (LBGI) [26]; CGM compliance; HbA1c ; total daily insulin dose (TDD). To evaluate glycaemic variability, standard deviation (SD), coefficient of variation (CV), and mean amplitude of glucose excursions (MAGE) will be measured [27]. Maternal glycaemic outcomes will be evaluated in each trimester and at the different time points during pregnancy, and will be compared with baseline and between both randomisation groups.

- Safety outcomes

Number and duration of hypoglycaemic episodes [time spent  < 3.5 mmol/L (< 63 mg/dL) and < 2.8 mmol/L (< 50 mg/dL)], nocturnal and/or severe hypoglycaemic episodes, and other adverse events such as diabetic ketoacidosis (DKA).

- Patient-reported outcomes

The following questionnaires will be completed at the different study visits:

1. A self-designed questionnaire on general habits and socio-economic background previously used in the Belgian Diabetes in Pregnancy Study (BEDIP-N) study to extensively collect information on socio-economic status and habits [28];
2. The Hypoglycaemia Fear Survey II (HFS-II) consisting of a 10‐item ‘behaviour’ subscale that measures behaviour involved in avoidance and overtreatment of hypoglycaemia and a 13‐item ‘worry’ subscale that measures anxiety and fear surrounding hypoglycaemia with higher scores indicating higher fear of hypoglycaemia [29];
3. The 36-Item Short Form Health Survey (SF-36) includes a set of generic, coherent, and easily administered quality of life measures that is validated for use in the maternity context [30];
4. The 20-item Centre for Epidemiologic Studies-Depression (CES-D) questionnaire which is validated in pregnancy to asses symptoms of clinical depression over the past seven days and consists of 20 items with each item being scored between 0 (rarely or not applicable) and 3 (almost all the time applicable) on a four-point Likert scale, resulting in a total score within the range 0–60, with a score of ≥ 16 being suggestive for clinical depression [31];
5. The Problem Areas in Diabetes-short form (PAID-5) contains items assessing fear, depressed mood and the demands of living with diabetes [32];
6. The Food Frequency questionnaire (FFQ) validated for the Belgian population includes questions on frequency and portion size of consumed foods and beverages [33];
7. The diabetes treatment satisfaction questionnaire (DTSQ) status which measures satisfaction with diabetes treatment regimens in people with diabetes [34].
8. The DTSQ change which has been developed to overcome potential ceiling effects (where respondents score maximum or near-maximum satisfaction at baseline and can show little or no improvement at follow-up) [35].

In addition, hypoglycaemia awareness status at each study visit will be determined by the Gold questionnaire, in which an individual’s experience in detecting hypoglycaemic events is scored from 1 (always aware) to 7 (never aware) in a Likert-type scale [36].

- Pregnancy outcomes

Obstetric outcomes: gestational weight gain; maternal hypertensive disorders including worsening of pre-existing hypertension, gestational hypertension, preeclampsia [≥ 20 weeks of gestation: new onset of hypertension and proteinuria or the new onset of hypertension and significant end-organ dysfunction with or without proteinuria (dipstick ≥ 2+, ≥ 0.3 g protein/24 hours or ≥ 30 mg/dL protein in spot urine or spot urine protein/creatinine ratio ≥ 30 mg protein/mmol creatinine)], and eclampsia (one or more generalized convulsions and/or coma in the setting of preeclampsia and in the absence of other neurologic conditions); other pregnancy complications [including Haemolysis, Elevated Liver enzymes and Low Platelets (HELLP) syndrome, polyhydramnios, and oligohydramnios]; hospital admissions and length of hospital stay (postpartum included); intra-uterine growth restriction (IUGR); fetal malformation; pregnancy duration; preterm delivery (< 37 weeks); type of labour [spontaneous, induced or planned caesarean section (C-section)]; mode of delivery (vaginal, instrumental, planned C-section or emergency C-section) and indications; miscarriage (< 20 weeks), stillbirth (fetal demise ≥ 20 weeks), and neonatal death (< 1 month after delivery); umbilical cord blood analysis including measurement of C-peptide.

Neonatal outcomes: sex; birth weight and percentile; macrosomia (> 4 kg); incidence of LGA, gestational age adjusted birth weight > 97th percentile, and small for gestational age (SGA), both also adjusted for infant’s sex and parity [37]; 10 min Apgar score; shoulder dystocia; birth trauma; congenital malformations; neonatal respiratory distress syndrome [at least four hours of respiratory support with supplemental oxygen, continuous positive airway pressure (CPAP), or intermittent positive-pressure ventilation during the first 24 h after delivery]; neonatal hypoglycaemia (glycaemia < 2.2 mmol/L or < 40 mg/dL or need for intravenous dextrose); neonatal jaundice (hyperbilirubinemia, need for phototherapy); duration and indication for admission on the NICU (admission defined as > 24 h); sum of skinfolds; neonatal fat mass; breastfeeding. These outcomes are included both as individual and as a composite outcome consisting of pregnancy loss (miscarriage, stillbirth or neonatal death), LGA, shoulder dystocia, birth trauma, respiratory distress, neonatal hypoglycaemia, and NICU admission.

- Health economic outcomes

The health economic evaluation of the HCL system (intervention) compared to SoC will be performed applying a cost-utility analysis consisting of two phases: (1) a trial-based economic evaluation, a cost-utility analysis of the intervention versus control alongside the RCT (including data on pregnancy outcomes), and (2) a model-based cost-utility analysis to predict costs and health effects of the intervention versus control on the longer term (including longer term health of the offspring). In both phases, health effects will be expressed as quality-adjusted life years (QALY) as recommended by the ISPOR-guidelines ‘Good research practices for cost-effectiveness alongside clinical trials’ [38]. Data on health-related quality of life will be collected using the SF-36 questionnaire [30]. Medical consumption and related costs will be collected as well as costs linked to the intervention. The analysis will be performed from a societal perspective, meaning that direct and indirect medical costs will be included.

### Recruitment and eligibility

Participants will be recruited from 11 endocrinology departments in Belgium (four university centres: University Hospitals Leuven, University Hospital Brussels, Ghent University Hospital, and University Hospital Saint-Luc; and seven large non-university hospitals: Imelda Hospital Bonheiden, OLV Hospital Aalst, Vitaz Campus Sint-Niklaas Moerland, General Hospital Groeninge Kortrijk, General Hospital Sint-Jan Brugge, General Hospital Delta Campus Rumbeke, and General Hospital Turnhout Campus Sint-Jozef) and one university centre in the Netherlands (Amsterdam University Medical Centres location AMC). The planned recruitment period is maximum two years. Written informed consent will be obtained at each site before any study-related activities are performed. Participants will be eligible if they fulfil the following criteria:

#### *Inclusion criteria*

- Women with T1D diagnosis of at least one year.
- Age 18–45 years.
- Singleton pregnancy up to 11 weeks and 6 days confirmed by β-hCG in blood and/or ultrasound.
- Intensive insulin treatment approved for use in pregnancy (MDI or CSII).
- Baseline HbA1c ≤ 86 mmol/mol (≤ 10%).
- Dutch, French or English speaking and understanding and e-mail access.

#### *Exclusion criteria*

- Use of a HCL system as closed-loop (allowed to use this system in open-loop).
- Multiple pregnancy.
- A physical or psychological disease likely to interfere with the conduct of the study according to evaluation by the treating physician.
- Medications known to interfere with glucose metabolism (intermittent use of prophylactic steroids for fetal lung maturation allowed).
- Insulin TDD ≥ 1.5 units/kg.
- Known allergy to adhesives for infusion set and/or CGM.

### Randomisation

Randomisation will be performed centrally by software and a randomisation algorithm developed by I Biostat. Participants will be randomised 1:1 to either the 780G (intervention) or SoC (control) group. Deterministic minimisation of variation will be used based on the following baseline characteristics: study centre, baseline HbA1c (locally determined at screening) stratified according to HbA1c < 53 vs. ≥ 53 mmol/mol (< 7 or ≥ 7%), and method of insulin delivery (MDI or insulin pump) [39].

### Groups

#### *Intervention*

Participants allocated to the intervention will be switched from their current diabetes therapy to the MiniMed™ 780G (Medtronic) HCL system. It received approval by the European Medicines Agency (EMA) in May 2020 and is currently under review for Food and Drug Administration (FDA) approval. This HCL system includes Proportional-Integral-Derivative (PID) technology with insulin feedback (the most advanced SmartGuard™ technology) consisting of the 780G insulin pump and Accu-Chek Guide Link meter with Guardian™ Link (3) transmitter and Guardian™ Sensor (3) or newer Guardian™ 4 transmitter and Guardian™ 4 sensor. The system allows for a glycaemic target at 5.5, 6.1 or 6.7 mmol/L (100, 110 or 120 mg/dL, respectively). To achieve tight glycaemic control in pregnancy, the following settings will be recommended in the study: glycaemic target set at 5.5 mmol/L (100 mg/dL) and active insulin time (AIT) set at 2 h. These settings may be adjusted during the study according to individual needs. In addition, the following recommendations will be made to limit the risk of postprandial hyperglycaemia: (1) optimise the insulin to carbohydrate ratios (ICR) by lowering the ratios as much as possible; (2) if further reduction of the ICR is impossible (due to occurrence of safe meal bolus, i.e. a bolus administered by the system which is lower than calculated based on the ICR due to prediction of an elevated risk of hypoglycemia), further reduction of carbohydrate intake with meals will be advised; (3) add fake carbs for a correction bolus in between meals or with meals. The amount and frequency of adding fake carbs will be registered at each visit at the diabetes clinic.

#### *Control*

Usual SoC was selected as control since RCTs have shown that both MDI and insulin pump therapy are equally effective to manage T1D in pregnancy [14]. Participants randomised to the control group will continue their current therapy [MDI or CSII with self-monitoring of blood glucose (SMBG) or any type of CGM; sensor-augmented pump therapy (predictive stop before low or on low) or a HCL system used in open-loop].

### Safety

Safety parameters will be assessed and reported at all study visits in line with the Medical Device Regulation (EU) 2017/745 and the MDCG 2020-10/1 European guideline. All (serious) adverse events [(S)AEs] and device deficiencies (DDs) will be recorded on applicable standardized reporting forms and associated with the subject identification number, with additional detailed description of the event. SAEs and reportable DDs will be reported onwards to the sponsor, manufacturer, central Medical Ethics Review Committee and national competent authorities as required.

### Discontinuation of participation

In case of premature discontinuation [due to withdrawal of participant’s consent, a significant medical reason or start of medication that does not allow for normal glucose metabolism evaluation (intermittent use of prophylactic steroids for fetal lung maturation allowed), a change in participant’s condition which justifies discontinuation of treatment, and noncompliance (e.g. not attending out-patient clinics)], data on glycaemic control and pregnancy outcomes will be further collected if an additional informed consent is provided.

### Study visits and data collection

An overview of timing of enrolment, interventions and assessments in the study is provided in Table 1.

**Table 1:** Overview of timing of enrolment, interventions and assessments in the CRISTAL study

		STUDY PERIOD									
**TIMEPOINTS**		**Screening**	**Baseline run-in** (within 1 week after screening)	**Randomisation**	**Training 780G HCL system or masked CGM** (within 1 week after run-in)	**Study visit 1 ** (if screening < 8 weeks)	**Study visit 2**	**Study visit 3**	**Study visit 4**	**Study visit 5**	**Delivery ** **and early postpartum**
**ENROLMENT**	**Eligibility screen**	X									
	**Informed consent**	X									
	**Allocation**			X							
**INTERVENTIONS**	***(masked) CGM for 10 days***		X								
	***(masked) CGM for 21 days***					X	X	X	X	X	
	***780G HCL system***				X	X	X	X	X	X	X
**ASSESSMENTS**	***Outcomes collected from medical records***:										
	Demographic data	X									
	Medical and obstetrical history	X									
	***Clinical and biochemical outcomes***:										
	Physical examination	X				X	X	X	X	X	
	HbA1c	X				X	X	X	X	X	
	Future analyses of new bio-markers and metabolomics	X				X	X	X	X	X	
	CGM metrics		X			X	X	X	X	X	X
	(S)AEs and DDs		X			X	X	X	X	X	X
	Other relevant health data	X				X	X	X	X	X	X
	C-peptide										X
	Future analyses of new bio-markers and metabolomics in umbilical cord blood										X
	Sum of skinfolds										X
	Neonatal body fat mass										X
	***Patient-reported outcomes***:										
	Self-administered questionnaires	X				X	X	X	X	X	
	Data for health-economic evaluation	X				X	X	X	X	X	X
	Medication use	X				X	X	X	X	X	X

HCL hybrid closed-loop; CGM continuous glucose monitoring; HbA1c glycated haemoglobin; (S)AEs (serious) adverse events; DDs device deficiencies.

#### *Baseline run-in phase*

Baseline CGM data (from the Guardian™ 3 or 4 CGM) of all participants will be collected during ten days. Only participants with a glucose monitoring method other than the Guardian™ 3 or 4 CGM will be requested to wear a masked Guardian™ 3 CGM in addition to their current glucose monitoring method. When using a masked Guardian™ 3 sensor, SMBG will be required at least twice a day for retrospective CGM calibration purpose.

#### *Training*

Within one week after the run-in phase, participants randomised to the intervention group will receive structured education on use of the 780G HCL system (ambulatory or with a short hospitalisation in line with normal routine). Participants allocated to the control group will be educated on the use of the masked Guardian™ 3 CGM, if applicable.

#### *During the screening visit and different study visits*

Study visits will be planned for participants of both groups at different time points in pregnancy: around 9 (if screened < 8 weeks), 14, 20, 26, and 33 weeks. The last study visit at 33 weeks can be performed earlier at 31–32 weeks if preterm delivery is expected. At each visit, a physical examination (with measurement of weight, blood pressure, and once height at screening) and blood collection (to measure HbA1c and for long-term storage in the biobank to allow future analyses of new biomarkers and metabolomics) will be performed. The glucose monitoring and insulin therapy data will be collected and reviewed to adjust the therapy, if needed. Differences in glycaemia between both groups will be evaluated by similar (masked) CGM data collected during 21 days at 9–12 (if screened < 8 weeks), 14–17, 20–23, 26–29, and 33–36 weeks. In addition, self-administered questionnaires, as listed above, will be completed at every study visit. Additional (tele)consultations can be performed according to need in between routine visits.

#### *Delivery and early postpartum*

At delivery and in early postpartum, based on the advice of the treating physician, the 780G HCL system can be continued, temporarily switched to open-loop or substituted by an insulin drip. If HCL is continued, increasing the glycaemic target to 6.1 or 6.7 mmol/L (110 or 120 mg/dL) and the ICR with at least 50% during the (end of the) dilation phase and delivery will be advised as a rapid decrease in insulin resistance is expected after delivery of the placenta [40]. After delivery, umbilical cord blood will be collected for measurement of C-peptide and storage in the biobank to allow future analyses of new biomarkers and metabolomics, if informed consent is provided. Neonatal skinfold thickness measurement will be performed within 72 h after birth, after giving informed consent, by trained study staff using a Harpenden skinfold caliper, as previously described in the HAPO study [41]. Skinfolds will be measured twice consecutively at the triceps, subscapular, and flank. The mean measured value at each site will be used to calculate the sum of skinfolds. Neonatal body fat mass will be determined according to a validated formula [42, 43].

### Statistics

#### *Sample size*

The sample size is determined to have about 90% power for the primary and three key secondary outcomes. To this purpose, at least 92 patients in total will be randomised. The sample size calculation was performed for these four outcomes separately, considering a Bonferroni correction for multiple testing to guarantee a 5% two-tailed family-wise significance level, and withholding the largest sample size. Calculations were performed for showing a time-averaged difference between the two groups over four longitudinal measurements [44]. A correlation of 0.6 was assumed between repeated measurements and a 20% loss to follow-up during the study was anticipated. For the primary outcome, the sample size was determined to detect an absolute between-group difference in mean TIR of 10% during pregnancy, assuming a SD of 13% for the TIR [22]. The power to show a difference of 1.1% between both groups for TBR, assuming a SD of 1.6% equals 91%. The power to show a difference of 1.6% between both groups for overnight TBR, assuming a SD of 2.5%, equals 87%. The power to show a difference of 15.2% between both groups in overnight TIR, assuming a SD of 18.4%, equals 97% [20]. After randomisation of approximately 50% of participants, a blinded sample size recalculation will be performed to re-estimate variances and correlations [45]. The sample size will be adjusted if needed to achieve the anticipated power.

#### *Data analysis*

Analyses will be performed according to intention-to-treat as well as per protocol. Clinical and demographic data at baseline will be summarised in a table using frequencies with percentages, mean values with SD or medians with the interquartile range (IQR). A linear mixed model for repeated measurements will be used for the primary outcome analysis, with TIR as response variable, and group, time point of measurement, stratification factors baseline HbA1c and method of insulin delivery, and finally baseline TIR as main effects, and a random effect of centre. A 1.25% significance level will be adopted. The mean difference between the groups will be presented with a 95% confidence interval. Using likelihood methods for estimation, this analysis provided valid results under a missing-at-random (MAR) drop-out pattern [46]. An analogous analysis method will be applied to the prespecified secondary outcome variables, with adjustment for the baseline value of the variable associated with the outcome, rather than baseline TIR. A similar analysis method will be applied to all longitudinally measured and continuous exploratory outcomes. Analysis of cross-sectional continuous outcomes will be performed by a two-sample two-sided t-test or a Mann-Whitney U test in case of skewed distributions. For comparing binary pregnancy outcomes between both groups, a Fisher exact test will be used, whereas a two-sample t-test or Mann-Whitney U test will be used for normally distributed or not-normally distributed continuous pregnancy outcomes, respectively. As a prespecified subgroup analysis, we plan to compare the primary and three key secondary outcomes between women who started the intervention early (screening < 8 weeks) versus later in pregnancy (screening ≥ 8 weeks).

### Trial management

This is an academic research trial. University Hospitals Gasthuisberg Leuven (UZ Leuven) is the sponsor and has responsibility for the overall management of the study.

#### *Data management*

Every site will be opened after a first initiation visit. Monitoring visits will be conducted to verify adherence of the participating sites to the protocol, standard operating procedures, Good Clinical Practice (ICH-GCP), and applicable regulatory and legal requirements, and to verify accuracy, completeness and verifiability of reported data. Monitoring in Belgian centres will be performed by an independent monitor of the clinical trial unit (CTU) of UZ Leuven and consists of a yearly interim monitoring visit (first monitoring visit within four months after the first recruited participant and yearly thereafter) and a close-out visit in each centre. Monitoring in Amsterdam University Medical Centres location AMC will be performed by an independent monitor of the local CTU with at least four monitoring visits on site and one remote visit. Each participant will receive a subject identification number to ensure confidentiality of the data. Data collected in this study will be referred to by subject identification numbers only. All obtained data will be entered and managed in the Research Electronic Data Capture (REDCap) platform. Encoded data will first be recorded by trained staff at each site on standardised paper worksheets and thereafter in REDCap according to applicable local guidelines. Glucose monitoring and insulin therapy data will be collected via manufacturer’s cloud software. Statistical analyses will occur in collaboration with the Centre of Biostatics and Statistical bioinformatics, KU Leuven.

#### *Trial steering committee*

A formal steering committee has been appointed to evaluate the progress and safety of the study, and to decide on whether the study should be paused or stopped according to the established formal rules. The steering committee consists of an independent chair, an independent methodologist and five researchers involved in the study.

Formal rules for pausing the study are:

- The occurrence of 5 hospitalizations for severe hypoglycaemia in participants from either the intervention or control group.
- The occurrence of 5 hospitalizations for DKA in participants from either the intervention or control group.
- A drop-out rate ≥ 40% in the first 20 participants in the study.

Formal rules for stopping the study are:

- The occurrence of 10 hospitalizations for severe hypoglycaemia in the intervention group.
- The occurrence of 10 hospitalizations for DKA in the intervention group.

## Discussion

This is the first large multicentre RCT evaluating the safety, efficacy, and cost-effectiveness of the widely used 780G HCL system compared to SoC in pregnant women with T1D starting in early pregnancy. Outside pregnancy, HCL systems have led to a paradigm shift in the management of T1D [24–26]. However, most commercially available HCL systems are currently not approved for use in pregnancy. This highlights the need for large RCTs to evaluate whether these HCL systems can be safely used in pregnancy and can improve glycaemic management in T1D in pregnancy.

The CRISTAL study aims to include a broad population of pregnant women with T1D, allowing for the use of all types of CGM systems. In addition, women with good glycaemic control can also participate in the study. This will allow for the inclusion of a representative population of pregnant women with T1D. Furthermore, we will commit to reporting all core outcome measures in the recently published COS for pregestational diabetes [24], which will facilitate the inclusion of data from our trial in future meta-analyses on the topic.

This study is important and innovative because we evaluate one of the most widely used commercially available HCL systems in people with T1D. Secondly, this is one of the largest ongoing studies investigating HCL systems in T1D in pregnancy. In addition, women are included in early pregnancy (< 12 weeks) and are followed up until the end of the postpartum hospitalisation to assess how fast the system can adapt to the non-pregnant state. If this study can show safe use and improved glycaemic management in this high-risk population, this will pave the way for the routine use of HCL systems in T1D in pregnancy.

## Data Availability

Requests for access to study data and stored samples will be considered where appropriate. Inquiries for data should be addressed to the principal investigator Prof. Katrien Benhalima.

## Abbreviations

T1D: type 1 diabetes
LGA: large-for-gestational age
TIR: time in range
CGM: continuous glucose monitoring
CSII: continuous subcutaneous insulin infusion
MDI: multiple daily injections
HCL: hybrid closed-loop
SoC: standard of care
RCT: randomized controlled trial
HbA1c: glycated haemoglobin
CE: Conformité Européenne
COS: core outcome set
TBR: time below range
TAR: time above range
LBGI: low blood glucose index
TDD: total daily dose
SD: standard deviation
CV: coefficient of variation
MAGE: mean amplitude of glucose excursions
DKA: diabetic ketoacidosis
BEDIP-N: Belgian Diabetes in Pregnancy Study
HFS-II: Hypoglycaemia Fear Survey II
SF-36 36-Item: Short Form Health Survey
CES-D 20-item: Centre for Epidemiologic Studies-Depression
PAID-5: Problem Areas in Diabetes-short form
FFQ: Food Frequency questionnaire
DTSQ: diabetes treatment satisfaction questionnaire
HELLP: Haemolysis, Elevated Liver enzymes and Low Platelets
IUGR: intra-uterine growth restriction
C-section: caesarean section
SGA: small-for-gestational age
CPAP: continuous positive airway pressure
NICU: neonatal intensive care unit
QALY: quality-adjusted life years
β-hCG: beta human chorionic gonadotrophin
EMA: European Medicines Agency
FDA: Food and Drug Administration
PID: Proportional-Integral-Derivative
AIT: active insulin time
ICR: insulin to carbohydrate ratios
SMBG: self-monitoring of blood glucose
(S)AE: (serious) adverse event
DD: device deficiency
HAPO: Hyperglycaemia and Adverse Pregnancy Outcome
IQR: interquartile range
MAR: missing-at-random
UZ: Leuven University Hospitals Gasthuisberg Leuven
ICH-GCP: International Conference on Harmonisation – Good Clinical Practice
CTU: clinical trial unit
REDCap: Research Electronic Data Capture

## References

[1] Evers IM, de Valk HW, Visser GH (2004). Risk of complications of pregnancy in women with type 1 diabetes: nationwide prospective study in the Netherlands. BMJ.

[2] Persson M, Norman M, Hanson U (2009). Obstetric and perinatal outcomes in type 1 diabetic pregnancies: a large, population-based study. Diabetes Care.

[3] Feig DS, Hwee J, Shah BR, Booth GL, Bierman AS, Lipscombe LL (2014). Trends in incidence of diabetes in pregnancy and serious perinatal outcomes: a large, population-based study in Ontario, Canada, 1996–2010. Diabetes Care.

[4] Macintosh MC, Fleming KM, Bailey JA, Doyle P, Modder J, Acolet D (2006). Perinatal mortality and congenital anomalies in babies of women with type 1 or type 2 diabetes in England, Wales, and Northern Ireland: population based study. BMJ.

[5] Battelino T, Danne T, Bergenstal RM, Amiel SA, Beck R, Biester T (2019). Clinical targets for continuous glucose Monitoring Data Interpretation: recommendations from the International Consensus on Time in Range. Diabetes Care.

[6] García-Patterson A, Gich I, Amini SB, Catalano PM, de Leiva A, Corcoy R (2010). Insulin requirements throughout pregnancy in women with type 1 diabetes mellitus: three changes of direction. Diabetologia.

[7] Murphy HR, Elleri D, Allen JM, Harris J, Simmons D, Rayman G (2012). Pathophysiology of postprandial hyperglycaemia in women with type 1 diabetes during pregnancy. Diabetologia.

[8] Feig DS, Donovan LE, Corcoy R, Murphy KE, Amiel SA, Hunt KF (2017). Continuous glucose monitoring in pregnant women with type 1 diabetes (CONCEPTT): a multicentre international randomised controlled trial. Lancet.

[9] Kristensen K, Ögge LE, Sengpiel V, Kjölhede K, Dotevall A, Elfvin A (2019). Continuous glucose monitoring in pregnant women with type 1 diabetes: an observational cohort study of 186 pregnancies. Diabetologia.

[10] Abell SK, Suen M, Pease A, Boyle JA, Soldatos G, Regan J (2017). Pregnancy outcomes and insulin requirements in women with type 1 diabetes treated with continuous Subcutaneous insulin infusion and multiple daily injections: Cohort Study. Diabetes Technol Ther.

[11] Kallas-Koeman MM, Kong JM, Klinke JA, Butalia S, Lodha AK, Lim KI (2014). Insulin pump use in pregnancy is associated with lower HbA1c without increasing the rate of severe hypoglycaemia or diabetic ketoacidosis in women with type 1 diabetes. Diabetologia.

[12] Rys PM, Ludwig-Slomczynska AH, Cyganek K, Malecki MT (2018). Continuous subcutaneous insulin infusion vs multiple daily injections in pregnant women with type 1 diabetes mellitus: a systematic review and meta-analysis of randomised controlled trials and observational studies. Eur J Endocrinol.

[13] Żurawska-Kliś M, Kosiński M, Kuchnicka A, Rurka M, Hałucha J, Wójcik M (2021). Continuous subcutaneous insulin infusion does not correspond with pregnancy outcomes despite better glycemic control as compared to multiple daily injections in type 1 diabetes - significance of pregnancy planning and prepregnancy HbA1c. Diabetes Res Clin Pract.

[14] Cummins E, Royle P, Snaith A, Greene A, Robertson L, McIntyre L et al. Clinical effectiveness and cost-effectiveness of continuous subcutaneous insulin infusion for diabetes: systematic review and economic evaluation. Health Technol Assess. 2010;14(11):iii-iv, xi-xvi, 1-181. 10.3310/hta14110 20223123

[15] Weisman A, Bai JW, Cardinez M, Kramer CK, Perkins BA (2017). Effect of artificial pancreas systems on glycaemic control in patients with type 1 diabetes: a systematic review and meta-analysis of outpatient randomised controlled trials. Lancet Diabetes Endocrinol.

[16] Leelarathna L, Choudhary P, Wilmot EG, Lumb A, Street T, Kar P (2021). Hybrid closed-loop therapy: where are we in 2021?. Diabetes Obes Metab.

[17] Boughton CK, Hovorka R (2021). New closed-loop insulin systems. Diabetologia.

[18] Murphy HR, Elleri D, Allen JM, Harris J, Simmons D, Rayman G (2011). Closed-loop insulin delivery during pregnancy complicated by type 1 diabetes. Diabetes Care.

[19] Murphy HR, Kumareswaran K, Elleri D, Allen JM, Caldwell K, Biagioni M (2011). Safety and efficacy of 24-h closed-loop insulin delivery in well-controlled pregnant women with type 1 diabetes: a randomized crossover case series. Diabetes Care.

[20] Stewart ZA, Wilinska ME, Hartnell S, Temple RC, Rayman G, Stanley KP (2016). Closed-Loop insulin delivery during pregnancy in women with type 1 diabetes. N Engl J Med.

[21] Stewart ZA, Yamamoto JM, Wilinska ME, Hartnell S, Farrington C, Hovorka R (2018). Adaptability of closed Loop during Labor, Delivery, and Postpartum: a secondary analysis of data from two randomized crossover trials in type 1 diabetes pregnancy. Diabetes Technol Ther.

[22] Stewart ZA, Wilinska ME, Hartnell S, O’Neil LK, Rayman G, Scott EM (2018). Day-and-night Closed-Loop insulin delivery in a Broad Population of pregnant women with type 1 diabetes: a randomized controlled crossover trial. Diabetes Care.

[23] Ozaslan B, Deshpande S, Doyle FJ, Dassau E (2021). Zone-MPC Automated insulin delivery algorithm tuned for pregnancy complicated by type 1 diabetes. Front Endocrinol (Lausanne).

[24] Kgosidialwa O, Bogdanet D, Egan AM, O’Shea PM, Newman C, Griffin TP (2021). A core outcome set for the treatment of pregnant women with pregestational diabetes: an international consensus study. BJOG.

[25] Beck RW, Bergenstal RM, Riddlesworth TD, Kollman C, Li Z, Brown AS (2019). Validation of Time in Range as an Outcome measure for diabetes clinical trials. Diabetes Care.

[26] Kovatchev BP, Cox DJ, Gonder-Frederick LA, Clarke W (1997). Symmetrization of the blood glucose measurement scale and its applications. Diabetes Care.

[27] Service FJ, O’Brien PC, Rizza RA (1987). Measurements of glucose control. Diabetes Care.

[28] Benhalima K, Van Crombrugge P, Verhaeghe J, Vandeginste S, Verlaenen H, Vercammen C (2014). The belgian diabetes in pregnancy study (BEDIP-N), a multi-centric prospective cohort study on screening for diabetes in pregnancy and gestational diabetes: methodology and design. BMC Pregnancy Childbirth.

[29] Gonder-Frederick LA, Schmidt KM, Vajda KA, Greear ML, Singh H, Shepard JA (2011). Psychometric properties of the hypoglycemia fear survey-ii for adults with type 1 diabetes. Diabetes Care.

[30] Petrou S, Morrell J, Spiby H (2009). Assessing the empirical validity of alternative multi-attribute utility measures in the maternity context. Health Qual Life Outcomes.

[31] Dalfrà MG, Nicolucci A, Bisson T, Bonsembiante B, Lapolla A, Quality of Life Italian Study Group (2012). Quality of life in pregnancy and post-partum: a study in diabetic patients. Qual Life Res.

[32] McGuire BE, Morrison TG, Hermanns N, Skovlund S, Eldrup E, Gagliardino J (2010). Short-form measures of diabetes-related emotional distress: the Problem Areas in Diabetes Scale (PAID)-5 and PAID-1. Diabetologia.

[33] Matthys C, Meulemans A, Van Der Schueren B (2015). Development and validation of general FFQ for use in clinical practice. Annals of Nutrition and Metabolism.

[34] Bradley C. The diabetes treatment satisfaction questionnaire. DTSQ. ed. Handbook of Psychology and Diabetes: A Guide to Psychological Measurement in Diabetes Research and Practice. Abingdon: Harwood Academic Publishers; 1994. p. 111 – 32.

[35] Bradley C (1999). Diabetes treatment satisfaction questionnaire. Change version for use alongside status version provides appropriate solution where ceiling effects occur. Diabetes Care.

[36] Gold AE, MacLeod KM, Frier BM (1994). Frequency of severe hypoglycemia in patients with type I diabetes with impaired awareness of hypoglycemia. Diabetes Care.

[37] Devlieger H, Martens G, Bekaert A, Eeckels R (2000). Standaarden van geboortegewicht-voor-zwangerschapsduur voor de vlaamse boreling. Tijdschrift voor geneeskunde.

[38] Ramsey SD, Willke RJ, Glick H, Reed SD, Augustovski F, Jonsson B (2015). Cost-effectiveness analysis alongside clinical trials II-An ISPOR Good Research Practices Task Force report. Value Health.

[39] Altman DG, Bland JM (2005). Treatment allocation by minimisation. BMJ.

[40] Draznin B, Aroda VR, Bakris G, Benson G, Brown FM, Freeman R (2022). 15. Management of diabetes in pregnancy: Standards of Medical Care in Diabetes-2022. Diabetes Care.

[41] Metzger BE, Lowe LP, Dyer AR, Trimble ER, Chaovarindr U, Coustan DR (2008). Hyperglycemia and adverse pregnancy outcomes. N Engl J Med.

[42] HAPO Study Cooperative Research Group (2009). Hyperglycemia and adverse pregnancy outcome (HAPO) study: associations with neonatal anthropometrics. Diabetes.

[43] Catalano PM, Thomas AJ, Avallone DA, Amini SB (1995). Anthropometric estimation of neonatal body composition. Am J Obstet Gynecol.

[44] Diggle HL. Analysis of Longitudinal Data. Oxford University Press; 2002.

[45] Kieser M, Friede T (2003). Simple procedures for blinded sample size adjustment that do not affect the type I error rate. Stat Med.

[46] Rubin DB (1976). Inference and Missing Data. Biometrika.

